# Secreted MicroRNA to Predict Embryo Implantation Outcome: From Research to Clinical Diagnostic Application

**DOI:** 10.3389/fcell.2020.586510

**Published:** 2020-09-22

**Authors:** Wei Zhou, Evdokia Dimitriadis

**Affiliations:** ^1^Department of Obstetrics and Gynaecology, The University of Melbourne, Parkville, VIC, Australia; ^2^Gynaecology Research Centre, The Royal Women’s Hospital, Parkville, VIC, Australia

**Keywords:** embryo implantation, non-invasive prediction, microRNAs, male factor, spent blastocyst culture medium, blood, uterine fluid, oocyte quality

## Abstract

Embryo implantation failure is considered a leading cause of infertility and a significant bottleneck for *in vitro* fertilization (IVF) treatment. Confirmed factors that lead to implantation failure involve unhealthy embryos, unreceptive endometrium, and asynchronous development and communication between the two. The quality of embryos is further dependent on sperm parameters, oocyte quality, and early embryo development after fertilization. The extensive involvement of such different factors contributes to the variability of implantation potential across different menstrual cycles. An ideal approach to predict the implantation outcome should not compromise embryo implantation. The use of clinical material, including follicular fluid, cumulus cells, sperm, seminal exosomes, spent blastocyst culture medium, blood, and uterine fluid, that can be collected relatively non-invasively without compromising embryo implantation in a transfer cycle opens new perspectives for the diagnosis of embryo implantation potential. Compositional comparison of these samples between fertile women and women or couples with implantation failure has identified both quantitative and qualitative differences in the expression of microRNAs (miRs) that hold diagnostic potential for implantation failure. Here, we review current findings of secreted miRs that have been identified to potentially be useful in predicting implantation outcome using material that can be collected relatively non-invasively. Developing non-invasive biomarkers of implantation potential would have a major impact on implantation failure and infertility.

## Introduction

Infertility affects a staggering one in six couples worldwide (Wilcox et al., 1988) and can be a devastating condition for couples, with the failure to conceive recognized as a leading cause of psychological distress, depression, low self-esteem, and domestic violence (Chachamovich et al., 2010; Cui, 2010). A major contributor to infertility is the failure of blastocysts to implant, accounting for >50% of all failed pregnancies (Craciunas et al., 2019). While *in vitro* fertilization (IVF) has increasingly assisted couples to conceive, success rates have stagnated as still, ∼50% of good quality blastocysts fail to implant (Gardner and Balaban, 2016). Implantation is a highly complex biological process that requires the coordination between a healthy embryo and a receptive endometrium. The process is initiated via fertilization of a healthy oocyte, which occurs in the Fallopian tube. During fertilization, the female reproductive tract serves as a natural selection system to guarantee that the best quality sperm reaches and fertilizes the oocyte (Ralt et al., 1991). Once fertilized, the zygote travels through the Fallopian tube and develops to the morula stage when it reaches the uterine cavity. The morula stage embryo continues to develop to the blastocyst stage in the uterine cavity before implantation (Norwitz et al., 2001). This can take up to 72 h within which time the embryo and the endometrium communicate via secreted and cell surface factors to prepare for the initial adhesion and attachment (Ashary et al., 2018). Once the outer layer of the embryo, namely, the trophectoderm firmly attaches to the endometrial luminal epithelium, it initiates implantation. Failure of firm adhesion leads to implantation failure.

Successful implantation is based on the cumulative success of the above events. Implantation can be affected by many factors including sperm and oocyte quality, early development of the embryo, the endometrium, and the reciprocal communication between blastocysts and endometrium. During an IVF clinical setting, embryo quality is generally scored via assessment of morphology, expansion and hatching, development of inner cell mass, and the formation of the trophectoderm layer (Giorgetti et al., 1995; Gardner et al., 2000). The transfer of embryos graded as good or “transferable” can improve implantation and pregnancy outcome (Giorgetti et al., 1995; Gardner et al., 2000). However, these morphological criteria do not necessarily correlate with implantation potential. Embryos with similar morphologically good scores assessed to be of transferable quality from aged women (>38 years) have a significantly lower pregnancy rate compared to those of younger women (<38 years) (Giorgetti et al., 1995). It is estimated that overall, 50% of good quality embryos fail to implant (Gardner and Balaban, 2016). In addition to scores based on morphology, pre-implantation genetic testing is also used in some IVF clinics. This testing requires the collection of trophectoderm cells to assess the ploidy of blastocysts and can reveal one of the many characters that may affect implantation. Another clinical approach to improve implantation success is via the assessment of endometrial receptivity. A current clinical test, called endometrial receptivity array, is used to evaluate whether the endometrium is in phase or receptive (Díaz-Gimeno et al., 2011). However, this method is invasive as it requires an endometrial biopsy and does not diagnose a disrupted or dysregulated endometrium, and while promising, there is still a need to develop non-invasive methods to recognize a disrupted endometrium (Díaz-Gimeno et al., 2011). In addition to the endometrium, sperm quality also can affect implantation. Current clinical analysis of sperm quality relies on the basic assessments of sperm morphology and motility, which do not necessarily reflect their capability in facilitating embryo development and implantation.

Despite the available tests, abnormalities in sperm, oocytes, disrupted endometrium, and embryo–endometrial interactions that contribute to implantation failure are not able to be effectively determined, and implantation failure remains a significant bottleneck for IVF treatment. To improve this, emerging work focuses on assessing biomarkers in samples that can be collected relatively non-invasively and examining whether they reflect the implantation potential. While many different classes of potential biomarkers have been proposed, microRNAs (miRs) stand out as promising biomarkers to determine the quality of sperm, oocytes, embryos, and endometrium that could be used to predict implantation outcome. miRs are small non-coding RNAs that regulate gene expression and protein production (Bushati and Cohen, 2007). Secreted or extracellular miRs are highly stable in body fluids, reflect disease states, and are easily detectable in a short time frame making them highly suitable for biomarker detection (Cuman et al., 2015). Emerging evidence strongly suggests that miRs regulate human embryo implantation (Paul et al., 2019). Recent studies support their use as non-invasive biomarkers for sperm, oocyte, and blastocyst quality, endometrial receptivity, and blastocyst–endometrial interactions (Cuman et al., 2015; Machtinger et al., 2017; Li et al., 2019; Abu-Halima et al., 2020). This review aims to discuss the use of miRs for screening blastocyst quality and implantation potential, focusing on using human samples that can be collected relatively non-invasively.

## Laboratory Identification of miRs With Translational Potential for Implantation Prediction

**FIGURE 1:**
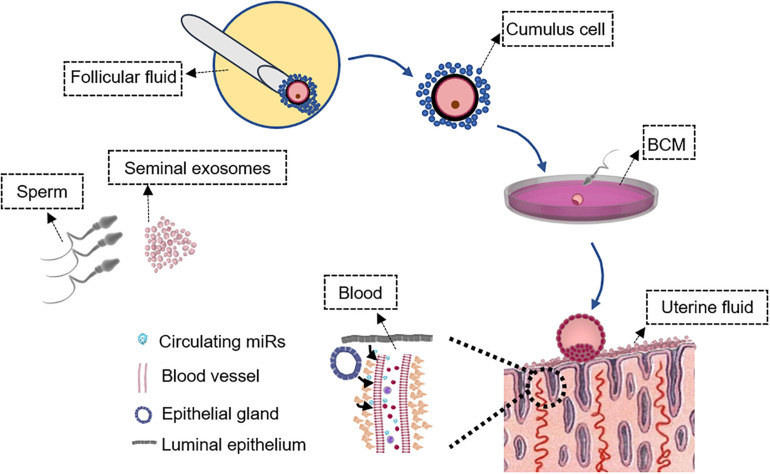
Schematic of samples that can be collected non-invasively during *in vitro* fertilization (IVF) treatment. Characterization of the seminal exosome and sperm microRNA (miR) profiles could determine sperm quality to predict the potential of embryo development and IVF outcome. Analysis of miR expression in follicular fluid and cumulus cells may indicate oocyte and embryo quality, and implantation potential from an embryo’s perspective. miR profiles in BCM also likely reflect embryo quality that may not be distinguished by morphology-based assessment. Endometrial cells release miRs in the blood and the expression levels of at least some endometrial miRs are reflected in the blood. Assessment of circulating miRs in the blood may predict endometrial receptivity and implantation outcome. Uterine fluid miR levels reflect “local” endometrial miR secretion and can be collected without compromising embryo transfer to provide information on endometrial receptivity. These samples are outlined. BCM, blastocyst culture medium.

A standard IVF treatment broadly requires egg retrieval, sperm collection, IVF, and embryo culture before transfer. The collection of cumulus cells and follicular fluid is possible during egg retrieval without affecting IVF (Figure 1). Analysis of gene and miR expression in follicular fluid and cumulus cells indicates oocyte and embryo quality, thus the implantation potential from an embryo’s perspective (Hamel et al., 2008; Fu et al., 2018). Embryos are generally cultured up to 5–6 days to reach the blastocyst stage before transfer (Figure 1). Embryos secrete specific profiles of miRs that may reflect their quality and implantation potential (Kropp et al., 2014; Capalbo et al., 2016). The endometrial epithelium secretes factors into the uterine cavity to regulate implantation and uterine fluid, or uterine lavage washings can potentially be used to detect miRs as biomarkers for the prediction of receptivity and implantation (Boomsma et al., 2009). Blood contains extracellular miRs with expression levels of some miRs positively correlating with endometrial levels and can potentially indicate whether the endometrium is dysregulated or receptive (Kresowik et al., 2014). Abnormalities in sperm contribute to blastocyst development and quality (Yuan et al., 2016), which could impact implantation. It has been shown in mice that sperm relays epigenetic information to the oocyte during fertilization and influences pre-implantation embryo and offspring development (Sharma et al., 2016). The development of non-invasive biomarkers has driven extensive research in this area with an overall aim to improve the success rate of implantation and IVF treatment.

## Cumulus Cells and Follicular Fluid

Cumulus cells are implicated in oocyte development and competence (Huang and Wells, 2010). In addition to interacting directly with the oocyte to facilitate maturation, cumulus cells are also bathed within the same follicular environment during oocyte maturation, thus may retain a footprint to reflect its quality and potential to form a viable embryo (Figure 1; Patrizio et al., 2007). It has been proven that cumulous cells are useful for non-invasive diagnosis of oocyte quality (Hamel et al., 2008; Devjak et al., 2016). Next-generation sequencing on human cumulus cells has revealed that miRs represent the major small RNA type, constituting as much as 71% of the total small RNAs (Xu et al., 2015). As a way of interaction, it has been shown that bovine cumulus cells and oocytes reciprocally affect the abundance of miRs in each cellular compartment (Abd El Naby, 2012), and these miRs readily control gene expression with extensive downstream functional implications. Gene expression studies on human cumulus cells have revealed transcripts that may be involved in oocyte maturation, implantation, and pregnancy with their regulatory miRs just beginning to be realized (Gasca et al., 2007; Hamel et al., 2008). During IVF treatment, cumulus cells are retrieved while still firmly attached to each oocyte, and as such, their collection can be sourced from individual oocytes and, thus provide an indication of the developmental potential for individual oocytes.

Follicular fluid is also collected during oocyte retrieval; however, unlike cumulus cells, follicular fluid normally collected during IVF stimulation is a pool from several oocytes, rather than a single oocyte to avoid multiple vaginal punctures. Despite this limitation, one study using pooled follicular fluid from individual patients has identified differences in miR expression between groups with different pregnancy outcomes (Scalici et al., 2016). This study screened five miRs and identified that hsa-miR-29a expression in the follicular fluid could predict pregnancy outcome with a specificity of 53.5% and has a higher discrimination power compared to prediction using embryo morphology scores (Scalici et al., 2016). Another two investigations collected follicular fluid from a single follicle and used microarrays to screen miRs that were able to predict the difference between good and bad quality blastocysts. Although hsa-miR-663b has been identified as a common miR that is inversely related to good quality blastocysts (Machtinger et al., 2017; Fu et al., 2018), the blastocyst quality discrimination method used in the two studies is based on routine morphological assessment. Therefore, the use of hsa-miR-663b as a marker of good quality blastocysts can similarly be determined by routine morphological assessment and likely provides limited application potential to predict embryo implantation outcome. In a clinical IVF setting, generally, multiple oocytes are retrieved at one time, and it is likely that they differ in quality and potential to develop into viable blastocysts. This may limit the extensive application of follicular fluid as it cannot be used to evaluate miRs released by individual oocytes.

## Seminal Plasma and Sperm

Defective sperm function is widely acknowledged as a major contributor to infertility. Under physiological conditions, the sperm acquires functional competence during their transit through the epididymis and female reproductive tract. Both biophysical and biochemical changes occur along this journey, eventually culminating in the ability of sperm to undergo an acrosome reaction, recognize the oocyte, and contribute to embryo development (Zhou et al., 2018). A number of recent studies in mice have provided evidence that uptake of miRs from the epididymal luminal environment endows the sperm with the capability to contribute to the early embryonic development and, thus, implantation upon delivery to the oocyte (Yuan et al., 2016; Conine et al., 2018, 2019). miR profile comparisons between mouse caput and cauda sperm have identified 27 miRs that are specifically enriched in cauda sperm, compared to caput sperm (Nixon et al., 2015; Sharma et al., 2018). Microinjection of cauda sperm-enriched miRs into caput-derived embryos rescue gene expression defects before implantation in mice (Conine et al., 2019). Further investigation has identified an epididymosome-dependent mechanism for the selective delivery of miRs into the sperm during their transit in the epididymis in mice (Reilly et al., 2016; Trigg et al., 2019; Zhou et al., 2019). In humans, differences in miR expression profiles have been recorded in both seminal plasma and sperm relative to different embryo qualities and pregnancy outcomes (Mokánszki et al., 2019; Abu-Halima et al., 2020; Xu et al., 2020). miR sequencing analysis on sperm samples grouped according to different embryo qualities has identified higher expression levels of hsa-miR-191 in the sperm group with better embryo developmental outcome (Xu et al., 2020). hsa-miR-19b-3p has a lower expression in sperm that is associated with a successful pregnancy outcome (Abu-Halima et al., 2020). Another recent study selected 11 spermatogenesis-related miRs and revealed that hsa-let-7a, hsa-miR-7-1-3p, hsa-miR-141, hsa-miR-200a, and hsa-miR-429 were significantly elevated, while hsa-miR-15b, hsa-miR-34b, and hsa-miR-122 were significantly downregulated in both seminal plasma and sperm of infertile male patients with impaired sperm production, compared to males with normal fertility (Mokánszki et al., 2019). Seminal plasma has been identified with an enriched population of epididymosome-like vesicles, namely, seminal exosomes (Vojtech et al., 2014). A limitation of using seminal exosomes is that they represent a mixed population of extracellular vesicles originating not only from the epididymis but also from the prostate and seminal vesicles (Rolland et al., 2013), Whether miR profiles in these vesicles correlate with sperm quality requires investigation. Nevertheless, seminal exosomes have been implicated in the transfer of cargo to sperm, which promotes their motility, ability to capacitate, and complete acrosomal exocytosis, therefore, affecting sperm quality (Tompkins et al., 2015). In addition, exosomes isolated from seminal plasma can modulate the immune response and gene expression changes in the female reproductive tract (Robertson and Sharkey, 2016; Bai et al., 2018), which eventually facilitate implantation and pregnancy in humans. Such functions are at least mediated via seminal exosome-carried miRs (Machtinger et al., 2016), and like epididymosomes, seminal exosomes carry distinctive profiles of miRs (Vojtech et al., 2014). Improved characterization of the seminal plasma and sperm miR profiles could not only be beneficial in terms of uncovering the causative basis of male gamete dysfunction but also for the provision of urgently needed biomarkers of sperm quality to reliably predict the outcome of IVF treatments.

## Spent Blastocyst Culture Medium

In an IVF setting, a fertilized oocyte is generally cultured *in vitro* for up to 5–6 days to the blastocyst stage before transfer. Spent culture media can be collected during media change without affecting embryo quality. It has been demonstrated that over 96% of miRs present in the spent culture media originate from the trophectoderm and can be consistently detected after blastulation under IVF culture conditions (Capalbo et al., 2016). It is tempting to speculate that blastocyst-secreted miRs participate in the regulation of trophectoderm–endometrial luminal epithelial interactions therefore implantation. In keeping with this notion, it has been identified that embryos with different implantation outcomes (implanted versus non-implanted) secrete different profiles of miR into the culture medium (Cuman et al., 2015; Borges et al., 2016; Capalbo et al., 2016). Increased expression of hsa-miR-142-3p and decreased expression of hsa-miR-20a and hsa-miR-30c have been identified in non-implanted blastocyst culture medium (BCM), compared to implanted BCM (Table 1; Borges et al., 2016; Capalbo et al., 2016). Further, microarray screens have identified a list of miRs exclusively detected in either implanted or non-implanted BCM (Table 1; Cuman et al., 2015; Capalbo et al., 2016). miR profiles in the BCM also likely reflect embryo quality and overall IVF outcome, as summarized in Table 1 (McCallie et al., 2010; Rosenbluth et al., 2014; Abu-Halima et al., 2020).

**TABLE 1:** Identified microRNAs (miRs) in blastocyst culture medium (BCM) with diagnostic potential.

**Total number of miRs examined**	**miR expression**	**References**
12 miRs	BCM from polycystic ovaries: Hsa-let-7a ↓, hsa-miR-24 ↓, hsa-miR-92 ↓, hsa-miR-93 ↓, hsa-miR-19a ↓, hsa-miR-19b ↓	McCallie et al., 2010
377 miRs	Non-implanted BCM: Hsa-miR-20a ↓, Hsa-miR-30c ↓	Capalbo et al., 2016
	Only detected in implanted BCM: Hsa-miR-220, hsa-miR-146b-3p, hsa-miR-512-3p, hsa-miR-34c, hsa-miR-375	
754 miRs	Failed IVF: Hsa-miR-191 ↑, hsa-miR-372 ↑, hsa-miR-645 ↑	Rosenbluth et al., 2014
7 miRs	Non-implanted BCM: Hsa-miR-142-3p ↑	Borges et al., 2016
784 miRs	Non-implanted group exclusively: Hsa-miR-374b-3p, hsa-miR-518c-3p, hsa-miR-126-3p, hsa-miR-361-5p,	Cuman et al., 2015
	hsa-miR-29b-2-5p, hsa-miR-516b-5p, hsa-miR-371a-5p, hsa-miR-372, hsa-miR-518a-3p, hsa-miR-149-5p, hsa-miR-571,	
	hsa-miR-943, hsa-miR-937-3p, hsa-miR-761, hsa-miR-106b-3p, hsa-miR-182-3p, hsa-miR-624, hsa-miR-661-5p,	
	hsa-miR-515-5p, hsa-let-7b-3p, hsa-miR-577, hsa-miR-1912	
	Implanted group exclusively: Hsa-miR-23a-3p, hsa-miR-570-3p, hsa-miR-485-3p, hsa-miR-572, hsa-miR-26b-5p,	
	hsa-miR-150-5p, hsa-miR-744-5p, hsa-miR-874, hsa-miR-24-2-5p, hsa-miR-300, hsa-miR-619, hsa-miR-208a,	
	hsa-miR-612, hsa-miR-26b-3p, hsa-miR-632, hsa-miR-362-3p, hsa-miR-543, hsa-miR-380-5p, hsa-miR-638	
372 miRs	High quality embryo: Hsa-miR-320a ↑, hsa-miR-15a-5p ↑, hsa-miR-21-5p ↓, hsa-miR-29a-3p ↓	Abu-Halima et al., 2020
	Negative pregnancy: Hsa-let-7a-5p ↑, hsa-miR-19b-3p ↓	

A previous study has proposed that while the pre-implantation embryo is in the uterine cavity, it packages regulatory miRs into extracellular vesicles (Ashary et al., 2018). They further propose that the packaged miRs are taken up by the endometrial luminal epithelial cells and alter their function to prepare for implantation. Incubation of primary human endometrial epithelial cells (HEECs) with BCM collected from embryos that were implanted increases their adhesive capacity to trophoblast cell line-formed spheroids (Cuman et al., 2013). Other notable examples include hsa-miR-661, which is exclusively secreted by blastocysts that fail to implant (Cuman et al., 2015). Secreted hsa-miR-661 from non-implanted BCM is taken up by HEECs and reduces their adhesion to trophoblast cell-formed spheroids (Cuman et al., 2015). A recent study also demonstrates that incubation of HEECs with BCM from embryos that implanted, compared to embryos that did not implant during IVF, leads to a substantial change in the expression of long non-coding RNAs in the HEECs (Takamura et al., 2019). PTENP1 is one of the most decreased long non-coding RNAs in HEECs after being treated with BCM from embryos that fail to implant (Takamura et al., 2019). Functionally, knockdown of PTENP1 impairs HEEC adhesion via a miR-dependent mechanism to downregulate gene targets essential for receptivity (Takamura et al., 2019).

The implanted and non-implanted embryos from which BCM was collected had an indistinguishable morphology based on currently available assessment of embryo quality. Thus, miRs in the BCM may serve as promising non-invasive biomarkers to improve the diagnostic accuracy of embryo quality and implantation potential. An obvious challenge to achieve this is to determine which cohorts of miRs are present in BCM samples that correlate with implantation outcome, in particular, regardless of embryo culture conditions. Although some miRs such as hsa-miR-19b-3p and hsa-miR-372 have been identified in at least two independent studies (Table 1), the comparison of secreted miRs in BCM with different implantation outcomes has demonstrated a generally inconsistent result among different studies (McCallie et al., 2010; Rosenbluth et al., 2014; Cuman et al., 2015; Borges et al., 2016; Capalbo et al., 2016; Abu-Halima et al., 2020). Contributing factors to this inconsistency include diverse embryo culture conditions in different IVF clinics, unstandardized protocols, manual effects on RNA isolation, and miR detection (Belandres et al., 2019). In addition, an obvious confounder of associating miRs in BCM with failed implantation outcome is the potential effects of the endometrium. A failed implantation group from which BCM was collected could be due to poor embryo quality, dysregulated endometrium, or altered receptivity window. The communication between an embryo and endometrium remains a “black box,” and it is perhaps notable that not all secreted miRs are taken up by endometrial luminal epithelial cells to regulate implantation. For future diagnostic purposes, it is necessary to identify which miRs are taken up by endometrium and their actions on the endometrium. A panel of miRs will likely to be included, with the functional consequence of each individual miR on implantation being confirmed in ideally both humans (*in vitro*) and preclinical animal models (*in vivo*). Detailed functional studies have only covered a small proportion of miRs identified so far (Table 2; Chu et al., 2015; Cuman et al., 2015; Kang et al., 2015; Kottawatta et al., 2015; Vilella et al., 2015; Zhang et al., 2015; Cai et al., 2016; Chen et al., 2016; Zheng et al., 2017; Sirohi et al., 2018; Winship et al., 2018; Balaguer et al., 2019; Griffiths et al., 2019; Takamura et al., 2019).

**TABLE 2:** miRs that regulate embryo implantation.

**miR**	**Phenotype**	**Target (s)**	**Affect implantation in animal model**	References
Hsa-miR-200c	Overexpression of miR-200c in RL95-2 and Ishikawa cells impair receptive ability *in vitro*	FUT4↓	Yes (mouse)	Zheng et al., 2017
Hsa-miR-661	Secreted by non-implanted embryo and transferred to HEECs to affect their adhesive capacity *in vitro*	PVRL1↓	Not available	Cuman et al., 2015
	Overexpression of miR-661 in HEECs *in vitro* impairs adhesion	MDM2↓	Not available	Winship et al., 2018
Hsa-miR-181a	Inhibition of miR-181a compromises human endometrial stromal cell decidualization *in vitro*	KLF12↓	Yes (mouse)	Chu et al., 2015; Zhang et al., 2015
Hsa-miR-212	hCG stimulates miR-212 expression in Ishikawa cells to favor spheroid adhesion *in vitro*	OLFM1↓, CTBP1↓	Not available	Kottawatta et al., 2015
Hsa-miR-145	Overexpression of miR-145 in Ishikawa cells affects mouse embryo attachment *in vitro*	IGF1R↓	Not available	Kang et al., 2015
Hsa-miR-30d	Human endometrial secreted miR-30d is taken up by mouse pre-implantation embryo and increases embryo adhesion *in vitro*	*Itgb3*↑, *Itga7*↑, *Cdh5*↑	Yes (Mouse)	Vilella et al., 2015; Balaguer et al., 2019
	Overexpression of miR-30d in Ishikawa cells facilitates adhesion *in vitro*	*SNAI1*↓	Yes (Mouse)	Cai et al., 2016; Balaguer et al., 2019
Hsa-miR-125b	Overexpression of miR-125b in HEECs inhibits cell migration and invasion *in vitro*	MMP26↓	Yes (Mouse)	Chen et al., 2016
Hsa-miR-29c	Overexpression of miR-29c in HEECs impairs adhesion *in vitro*	COL4A1↓	Not available	Griffiths et al., 2019
Hsa-miR-590-3p	Overexpression of miR-590-3p in HEECs *in vitro* impairs adhesion	N/A	Not available	Takamura et al., 2019
Hsa-miR-140	Overexpression of miR-140 in RL95-2 endometrial epithelial cells impairs adhesion and spheroid outgrowth *in vitro*	N/A	Yes (Rat)	Sirohi et al., 2018

## Blood

MicroRNAs are also readily secreted into the blood. Circulating miRs are packaged in membrane-bound vesicles, attached to high-density lipoproteins or bound to RNA-binding proteins, which endow them with striking stability in the blood (Schwarzenbach et al., 2014). The human endometrium features a rich blood supply with the responsibility to provide an optimal environment to promote receptivity and implantation (Farrer-Brown et al., 1970). Endometrial cells may secrete/transport a number of miRs to the tissue site of action by way of the blood, and studies suggest that endometrial expression levels of at least some miRs are reflected in the blood (Kresowik et al., 2014; Di Pietro et al., 2018). Circulating miRs in the blood may be able to predict endometrial receptivity and implantation. A previous study used whole blood and paired mid-secretory phase endometrial tissue to determine whether circulating miRs could distinguish fertile from recurrent implantation failure patients (Rekker et al., 2018). miR-30a-5p was identified as differentially expressed in whole blood between the two groups; however, this difference was not reflected in the paired endometrial tissue (Rekker et al., 2018). One possible explanation is that blood cells express miRs (Jickling et al., 2014), which may mask endometrial tissue-secreted miRs. Recent work using paired serum and mid-secretory phase endometrium investigated five miRs and identified a positive correlation of hsa-miR-31 expression levels between serum and endometrial tissue (Kresowik et al., 2014). Alternatively, extracellular miR expression levels do not necessarily reflect cellular expression levels. Whether miR biomarkers have critical functions in endometrial receptivity also needs to be determined experimentally, as differentially expressed circulating miRs between women with normal fertility and infertility may not all have functional relevance in receptivity or implantation. Functional studies of the identified circulating and cellular miRs in receptivity and implantation models could provide evidence to support their potential application as biomarkers and treatment targets. For example, hsa-miR-200c expression is increased in the serum of infertility and abortion patients, compared to healthy women (Zheng et al., 2017). Functional analysis using both human endometrial cell lines and a mouse model demonstrates that hsa-miR-200c overexpression impairs endometrial cell receptivity in both species (Zheng et al., 2017).

The obvious challenge to predict implantation outcome using miRs in the blood will be to distinguish the endometrial secreted miRs from miRs secreted by other tissues. Of note, the process of embryo implantation somewhat resembles that of cancer cell metastasis. Both processes share some of the cellular mechanisms in cell adhesion, invasion, and angiogenesis (Murray and Lessey, 1999). miRs such as hsa-miR-29c (Griffiths et al., 2019) and hsa-miR-125b (Chen et al., 2016) that are dysregulated in the endometrium from infertile women are also associated with gastric and endometrial cancers (Shang et al., 2012; Wang et al., 2019). Cancer cells releasing miRs into the blood may confound the detection of miRs secreted by the endometrium. In this regard, an important feature of the endometrium is that it regenerates itself at each menstrual cycle. The endometrium is only receptive to an implanting embryo within a very short window in the mid-secretory phase (Ashary et al., 2018). Such a functional switch is mediated by coordinated changes of miR expression (Vilella et al., 2015). These phase-dependent changes, in turn, may endow endometrial-secreted miRs with unique cycle-dependent expression fingerprints that can be used to distinguish from the background of other potential tissue-secreted miRs. This theory is evidenced by a previous study comparing miR expression between the proliferative phase and mid-secretory phase in paired serum and endometrial tissue from fertile women. hsa-miR-31 has been identified as a potential biomarker that is elevated in both serum and endometrium in the mid-secretory phase, compared to the proliferative phase (Kresowik et al., 2014). It is also essential to investigate appropriate controls from different pathologies as comparative groups. The predictive application of blood miRs on implantation will likely be based on the multiple measurements of miR expression at different phases within a menstrual cycle.

Another challenge of using miR levels in the blood for biomarker purposes has been identified in cancer diagnosis. A previous study selected 79 solid cancer-circulating miR biomarkers and determined their expression levels in blood cells. Forty-six of the 79 miRs were highly expressed in the blood cells (Pritchard et al., 2012). Plasma isolated from the blood with different blood cell counts or hemolysis impacted the expression levels of select miRs (Pritchard et al., 2012). Inconsistency has also been observed between plasma and serum levels of miR between pregnant and non-pregnant patient groups after embryo transfer (Yang et al., 2018). To improve the accuracy of prediction, a panel of miRs is required, as has been proposed for cancer diagnosis (Madhavan et al., 2013). To achieve this, an investigation of miR levels from large cohorts of women with different etiologies of infertility and other pathologies is required for their potential use as biomarkers. We have previously identified a dysregulation of miR-processing machinery in the endometrium of a cohort of infertile patients, which would have an overall impact on miR secretion due to compromised miR processing within the cell (Loke et al., 2019). The miR secretion in this cohort may be different compared to other infertile cohorts caused by different etiologies. Identifying which miRs are responsible for ensuring endometrial receptivity is also required to determine whether the biomarkers may also be useful as treatment targets of dysregulated endometrial receptivity.

## Uterine Fluid

The uterine fluid is secreted by the human endometrium as an indirect approach to communicate with an embryo for the preparation of implantation. Compared to other body fluids, uterine fluid is a more “local” secretion and, thus, may provide direct information when assessing biomarkers for implantation. Detailed compositional analysis has revealed that uterine fluid contains miRs and proteins with changed profiles across the menstrual cycle (Scotchie et al., 2009; Ng et al., 2013). Functional analysis has proven that endometrial cells secreted miRs, such as hsa-miR-30d, that are taken up by the embryo via the trophectoderm and regulate adhesion *in vitro* (Vilella et al., 2015). Further investigation demonstrates that secreted miRs in the uterine fluid target an extensive of implantation-related genes (Ng et al., 2013). Of note, the miRs in the uterine fluid can be sourced from different endometrial cells and the blood. This can be determined via *in situ* hybridization on endometrial sections, like what has been done for protein via immunostaining (Hannan et al., 2010). Uterine fluid can be collected via either aspiration or lavage without compromising implantation (Hannan et al., 2012). To the best of our knowledge, however, most currently available studies on uterine fluid have focused on comparing the proteins between fertile and infertile patients (Hannan et al., 2010; Salamonsen et al., 2013). There are presently limited studies investigating the potential of using miRs in the uterine fluid as a diagnostic approach for implantation.

## Overall Challenges of Using Secreted miRs to Predict Implantation

Although miRs are highly desirable as non-invasive biomarkers to predict implantation, this field of research is somewhat confounded by a general inconsistency of miR expression levels across different studies. It has been identified that a number of factors including RNA isolation and detection systems can contribute to this inconsistency. Recently published work from one laboratory, which used different commercial kits to isolate RNA, demonstrated that the recovery of RNA was variable between the commercial kits (El-Khoury et al., 2016; Wright et al., 2020). In addition, the selection of endogenous controls to normalize the target miR expression levels directly affects the results, and such importance has been neglected by some studies. For miR normalization, an ideal endogenous control should be stably expressed in the body fluid with minimal biological variation, and the expression should not change with different implantation outcomes. It is known that in body fluids, the expression of some cellular endogenous controls may vary between different samples bringing deviation in normalization. It is an essential first step to compare the expression variability of a number of endogenous control candidates in a given body fluid system and confirm their stability. This has not been conducted in some studies and may have contributed to the variability of miR expression. A workflow has been proposed to identify the best normalization control (Schwarzenbach et al., 2015). All these steps introducing impact factors require standardization before a solid conclusion can be drawn.

Adding to this challenge is the observation that inherent differences between women, together with different IVF protocols, may lead to differential expression patterns of miR in the human endometrium. A microarray study has identified that luteal support following controlled ovarian stimulation has a profound influence on the miR profile in the endometrium (Zhao et al., 2012). Specifically, progesterone supplementation is associated with a significant increase in miR expression in the endometrium compared to a no steroid supplementation group following controlled ovarian stimulation (Zhao et al., 2012). The findings are in accordance with a previous report identifying differential expression patterns of miR between natural and stimulated IVF cycles (Sha et al., 2011). In addition, patients receiving the same IVF treatment who have different serum progesterone levels have been identified to have different miR expression patterns in the endometrial tissue collected 6 days after oocyte retrieval (Li et al., 2011). Microarray analysis of the endometrium identified four miRs (hsa-miR-451, hsa-miR-424, hsa-miR-125b, and hsa-miR-30b) that were decreased in the high serum progesterone group (Li et al., 2011). The effects of controlled ovarian stimulation and luteal phase support need to be considered when comparing data from different studies.

## Conclusion

Measurement of miRs in the samples that can be collected without compromising embryo transfer in the same menstrual cycle opens new perspectives for the diagnosis of embryo implantation potential. Unfortunately, our understanding of the mechanisms of how miR dysregulation impacts implantation and how this accordingly affects miR secretion remains far from complete. Interpretation of research findings is confounded by unstandardized assessment of miRs in a given body fluid. Resolving these questions would have a major impact on biomarker development and clinical practice for reproductive clinicians and scientists. This includes optimizing the selection of embryos for transfer during IVF, improvement of implantation success rates, and the minimization of multiple pregnancies. It is likely that a combination of samples that can be collected either non-invasively or relatively non-invasively, as summarized in this review, will be useful to assess implantation potential at different stages of conceptus establishment and development. This relies on research to find miR biomarkers related to implantation regulation and the development of new technologies to improve miR detection. A few microfluidic devices have been developed recently with a larger capacity to include more miRs and reduce analysis time. Improved diagnosis of embryo implantation could have a profound effect on psychological and financial well-being on women and couples undergoing IVF treatment.

## Author Contributions

Both authors made substantial contributions to the conception of this review and the critical appraisal of the literature summarized herein, wrote the manuscript, and approved the final version of this article before submission.

## Conflict of Interest

The authors declare that the research was conducted in the absence of any commercial or financial relationships that could be construed as a potential conflict of interest.
